# The creation of “Ecosystem Core” hypothesis to explain ecosystem evolution

**DOI:** 10.1186/s12898-019-0251-y

**Published:** 2019-09-06

**Authors:** Kun Wang, Xiajie Zhai

**Affiliations:** 1 College of Grassland Science and Technology, China Agricultural University, Beijing, 100193 China; 2 Beijing Key Laboratory of Wetland Services and Restoration, Institute of Wetland Research, Chinese Academy of Forestry, Beijing, 100091 China

**Keywords:** Ecosystem, Evolution, Emergy, Life, Environment

## Abstract

**Background:**

Humans have dramatically changed natural ecosystems around the world as their capacity to manage their environment for multiple uses has evolved in step with agricultural, industrial and green revolutions. Numerous natural ecosystems have been replaced by various artificial or semi-artificial ecosystems, the ecosystem has changed. To a certain extent, this is ecosystem evolution. So far, there is no definite ecological theory about the mechanism for evolution of an ecosystem. Even though the discipline of community ecology has a relatively comprehensive and well-described theory of succession, at the different ecological research levels, is it the same mechanism for the community succession and ecosystem evolution? What is the factor that drives ecosystem evolution?

**Results:**

This paper puts forward the “Ecosystem Core” hypothesis to scientifically address the above problems. We define abiotic component of ecosystem as “Ecosystem Core” or “Resource Core”, which provides the foundation (matter and energy) for the existence and progress of organisms and should be the nucleus of an ecosystem. In this paper, we explain the basic meaning of this hypothesis, review its theoretical foundation, and provide a demonstration (based on emergy theory, which is an accounting tool that considers both the environmental and economic inputs that are directly or indirectly required by a process to generate a product and it measures real wealth, independent of financial considerations) of the hypothesis, and discuss the mechanism of ecosystem evolution. The “Ecosystem Core” hypothesis reveals the quantitative relationship between the energy input and ecosystem evolution.

**Conclusions:**

The input of artificial auxiliary energy is the direct cause of ecosystem evolution. Different combinations of natural and purchased emergy are coupled to maintain the same ecosystem under the different environmental conditions. When artificial energy enters the ecosystem, its role is similar to that of the microscopic particles that collide with the nucleus in the nuclear reaction, and after mutual reaction, the atom will form a new atomic structure, and for the ecosystem, a new form of resource composition and energy action will appear, and the corresponding species of life will change, then ecosystem complete its evolution.

## Background

Ecology encompasses multiple levels of research, from molecular level to landscape level. In the study of community ecology, a relatively well-described and comprehensive theory of succession has been formed [1]. Community succession is the process of community change following disturbance by natural or human disturbance where the community composition, especially the dominant species, changes. This is a phenomenon in which one community is replaced by another at different times in the same place. After the ecosystem concept was presented in the 1930s [2], scholars used systematic theory and a holistic view to study the process and phenomenon of life forming a more complete ecosystem theory. Discipline of ecosystem ecology is continuously improving, and has become the focal area of ecological research. Ecosystem degradation [3, 4], biodiversity loss [5, 6], eutrophication [7], biological invasion [8] or novel ecosystems (emerging ecosystems, result when species occur in combinations and relative abundances that have not occurred previously within a given biome [9]) and climate change [10] suggest that ecosystems are constantly changing due to natural and anthropogenic factors in the real world. An ecosystem is replaced by another ecosystem, which we call the ecosystem evolution. So far, there is no definite theory about the mechanism for evolution of an ecosystem which raises the following questions: is it the same mechanism for the ecosystem evolution and community succession? What is the factor that drives the evolution of the ecosystem? After years of attention and study on these scientific issues, we write this paper to propose the hypothesis of “Ecosystem Core”, in order to scientifically address these problems.

## Results and discussion

### The basic meaning of “Ecosystem Core” hypothesis

#### Interpretation of atomic theory

**Fig. 1:**
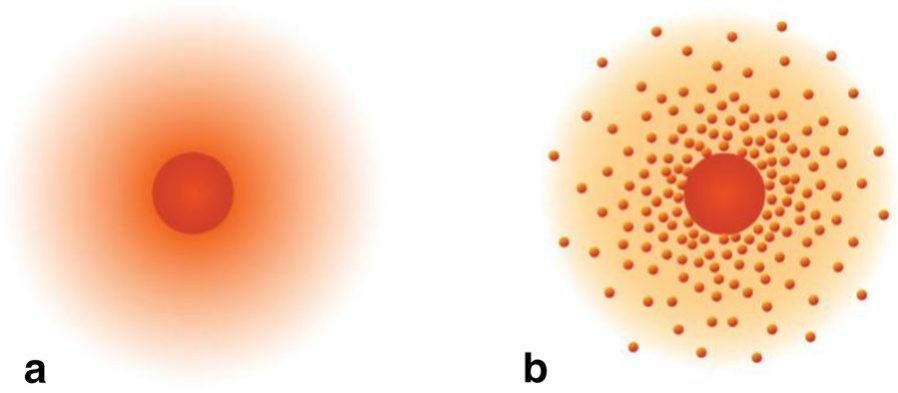
A modern depiction of atomic structure. **a** The darker the color, the higher the probability that an electron will be at that point. **b** In a two-dimensional cross section of the electron in a hydrogen atom, the more crowded the dots, the higher the probability that an electron will be at that point. In both (**a**) and (**b**), the nucleus is in the center of the diagram [11]

Classical physics shows that the atom is composed of a positively charged nucleus and negatively charged extranuclear electrons, which are attracted by positive and negative electricity to form a complete and stable atomic structure. The extranuclear electrons are in different orbits because of the different charges they carry. When an electron absorbs a certain amount of energy, it will jump from one level to another, and when the absorbed energy is sufficient to exceed the gravitational force between electrons and nuclear, the electrons will “escape”. The electron cloud and the atom nucleus are combined together by the charge, which make up the atom (Fig. 1).

#### The basic meaning of “Ecosystem Core” hypothesis

An ecosystem is a community of living organisms in conjunction with the nonliving components of their environment [12]. The abiotic components is essential for the existence and development of organisms and includes light, temperature, water, air, inorganic part of soil, etc. The organic part mainly refers to the relationship between organisms including plants, animals and microbes, which are subdivided into producers, consumers and decomposers. These biotic and abiotic components are linked together through matter cycles and energy flows [13]. Generally, there are certain kinds of organisms in what kind of environments exists, and the environment determines the existence of organisms. However, while organisms are adapting to the environment, they also have a transformative effect on the environment.

**Fig. 2:**
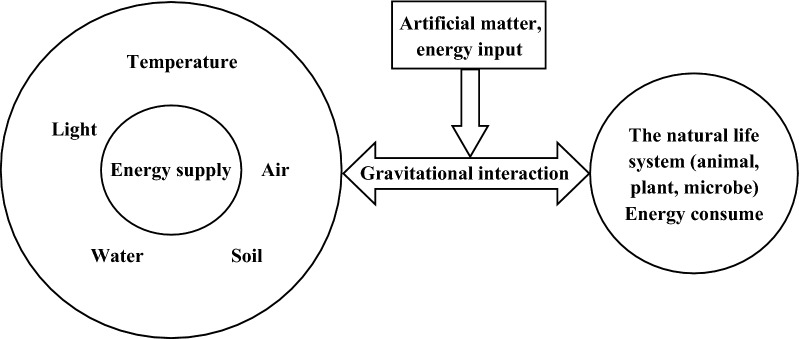
“Ecosystem core” hypothesis model

When compared with atomic structure, the relationship between organism and the environment in an ecosystem is similar. In the ecosystem, the abiotic component of ecosystem (resources) is similar to the atomic nucleus, we call it “Ecosystem Core or Resource Core”, and all kinds of abiotic components provide the matter and energy for the existence and development of the living creature, with positive electricity; biotic component of ecosystem (life) consumes energy, which is equivalent to extra-nuclear electrons, with negative electricity. Abiotic and biotic components of ecosystem are combined by the gravitational effect of energy. In fact, by using resources the living form is also maintaining and transforming the environment by shape shifting matter and energy, and forming a circulation state (Fig. 2).

**Fig. 3:**
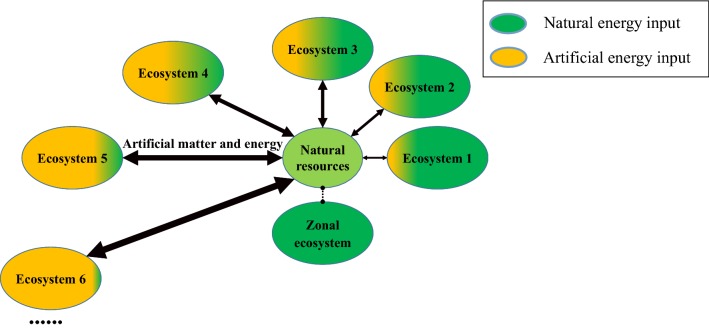
Conceptual model of ecosystem evolution

Under the natural condition without human disturbance, a specific ecosystem corresponds to a certain “Ecosystem Core”, and the emergence of a latitudinal and longitudinal distribution of vegetation on the earth is the concrete embodiment of this hypothesis. When human’s disturb a natural ecosystem by increasing energy inputs or changing the output of the system’ state, the ecosystem changes or becomes a different ecosystem resembling the electronic transition of an atom; when a disturbance far exceeds the energy provided by the “Ecosystem Core”, it transitions from a natural system to an entirely artificial intelligent ecosystem (e.g. soilless farming) that resembles an electron “escaping” in an atom (Fig. 3). In the evolution of the ecosystem, various ecological factors play a role together, leading to change in the structure and function of the system, and the vary of matter and energy are the main driving factors. That is, changing the input of matter and energy will change the natural living system, which is the mechanism of ecosystem evolution.

### Theoretical foundation of “Ecosystem Core” hypothesis

Matter cycling and energy flows are basis features and functions of any natural ecosystem, the inclusion of diverse organisms makes it an “ecosystem”. The law of conservation of matter and the law of conservation of energy are still the theoretical basis of the “Ecosystem Core” hypothesis. The principle of structure and function is the most essential characteristic of the ecosystem. Ecosystem structure reflects the organization of various abiotic and biotic pools that exchange energy and matter [14], which is the basis of the function. For example, community composition and distribution [15, 16], biodiversity [17, 18], and food webs [19]. Ecosystem function is the physical, chemical, and biological processes or attributes that contribute to the self-maintenance of the ecosystem, including energy flow, nutrient cycling, filtering, buffering of contaminants, and regulation of populations [19], which is the expression of the structure.

In nature, vegetation or plant communities are changing from time to time. In the same place, the replacement of the old plant community by a new plant community is called vegetation succession. Similarly, an ecosystem is replaced by another ecosystem, which we call the ecosystem evolution. The two are related and have distinct characteristics. Community ecology is concerned with the composition of life- the plant itself, such as species composition, productivity and biodiversity. It is the response of the plant itself to natural environmental factors or interference, for example, the diversity-stability debate [20]. On average, diversity give rise to stability, but it depends on factors including the intrinsic responses of species to environmental fluctuations, the speed at which species respond to perturbations and the strength of competition [21]; the ecosystem consists of the non-living environment and the living matter itself, and the life part contains animals, plants and microorganisms, and the relationship between them is very complex, and it is a comprehensive reflection of the ecosystem under the interference of natural or man-made. Therefore, the evolution of an ecosystem is more complex than community succession.

Succession is mainly caused by natural factors and human disturbance. If this succession occurs without external interference, it is natural succession. In general, natural ecosystems have their own unique structure and function types, although the structure and function are also changing. This alter is often only a fluctuation, or is considered a fluctuating balance [22]. Some directional changes may evolve over a long period. When there is no fundamental change of the environmental conditions in the ecosystem, especially in soil properties, if sufficient time is available, the biological community can be restored to a state similar to its natural condition [23]. In the present world, an ecosystem that has not been disturbed by human beings is very rare [24]. It is more common to find ecosystems that are continuously disturbed than non-disturbed ecosystems. Human interference encompasses the utilization, abandonment, transformation, reconstruction, and restoration of natural systems. These disturbances sometimes happen alone or they sometimes interfere with other disturbances. The human interference constitutes the main driving force of ecosystem change and it is comprised by the time, scale and intensity of a disturbance.

### Demonstration of “Ecosystem Core” hypothesis

On the planet we live on, in areas where the latitude and longitude are relatively definite, and the terrain is basically consistent, their light, precipitation, temperature, humidity, soil and biological systems can be regarded as the same. In accordance with the laws of nature, if there is no human disturbance, the ecosystem formed by them should belong to the same type. However, this situation is very rare today. Under the intense disturbance of human activities, it is very difficult to find the natural ecosystem that is undisturbed replaced by many ecosystem types coexisting, especially these ecosystems of man-made or various degrees of interference dominated the mainstream [4, 25]. So far, 12% of the earth’s land surface has been reclaimed as cropland [26], add deforestation, infrastructure, urban use and so on, with a total of 18–29% [27], which is already much different from the original ecosystem type. For example, the agro-pastoral ecotone of northern China was originally the grassland ecosystem of Eurasia, but with the large increase of population, people began to reclaim grassland in a large area, and then there are dozens of artificial and semi-artificial ecosystem types, including farmland, artificial forest, vegetable field, forage land, wetland, grazing land, vegetable greenhouses, and so forth. These ecosystems exist either for human economic purposes or are constrained by natural climate and soil conditions, coexist in a certain area and form a composite landscape that we think.

#### Coexistence mechanism of different ecosystem types under the same climate and soil conditions

Neutral theory and niche (construction) theory explain why species in a community can coexist [28–30]. However, it is not clear that coexistence mechanism of different ecosystem types under the same climate and soil conditions. Some studies of physical geography have shown that, without or with little human interference, a climate condition should correspond to a major top-level ecosystem type; and some auxiliary ecosystems can be found due to local topographic variations. It’s called monoclimax hypothesis [31]. Guyuan County of Hebei Province is a typical representative area of agro-pastoral ecotone in northern China. The county has a population of 260 thousands and a land area of 36.01 × 10^4^ ha, of which cultivated land accounts for 40%, natural grassland and woodland occupy 50%, and 10% are water surface, roads, dwellings and so on. We selected 12 of the major ecosystem types as our research subjects that including four land use types: commercial crop, field crop, artificial forage and grassland. The energy input analysis was carried out according to the emergy theory (it is a universal measure of real wealth of the work of nature and society based on a common basis, and can be described as the available energy of one kind previously required to be used up directly and indirectly to make the product or service [32]), as showed in Tables 1 and 2.

**Table 1:** Emergy input of main ecosystems in Guyuan County of Hebei Province, China Some data refer to Zhai et al. [33]

Ecosystem types	Ecosystems	Natural emergy input (× 10^14^ sej/ha/year)	Purchased emergy input (× 10^14^ sej/ha/year)	Sum of emergy input (× 10^14^ sej/ha/year)
Commercial crop	Potatoes	5.31	147.67	152.98
	Chinese cabbage greenhouse	5.31	146.36	151.67
	Spinach greenhouse	5.31	146.35	151.66
	Cabbage	5.31	101.04	106.35
Artificial forage	Oats	5.31	15.63	20.94
	Corn silage	5.31	12.09	17.40
Field crop	Naked oats	5.31	6.94	12.25
	Flax	5.31	6.90	12.21
	Wheat	5.31	6.89	12.20
Grassland	Free grazing	5.31	4.21	9.52
	Chinese leymus grassland	5.31	3.54	8.85
	Natural mowed grassland	5.31	3.53	8.84

**Table 2:** Comparative of main ecosystems in Guyuan County of Hebei Province, China Some data refer to Zhai et al. [33]

Ecosystem types	Ecosystems	Emergy investment ratio (EIR)	Emergy self-sufficiency ratio (ESR) (%)	Emergy yield ratio (EYR)	Environmental loading ratio (ELR)	Emergy sustainability index (ESI)
Commercial crop	Potatoes	27.81	3.47	1.04	31.00	0.0334
	Chinese cabbage greenhouse	27.56	3.50	1.04	30.73	0.0337
	Spinach greenhouse	27.56	3.50	1.04	30.73	0.0337
	Cabbage	19.03	4.99	1.05	21.25	0.0495
Artificial forage	Oats	2.94	25.36	1.34	3.38	0.3963
	Corn silage	2.28	30.52	1.44	2.64	0.5452
Field crop	Naked oats	1.31	43.34	1.76	1.56	1.1289
	Flax	1.30	43.51	1.77	1.55	1.1395
	Wheat	1.30	43.51	1.77	1.55	1.1398
Grassland	Free grazing	0.79	55.79	2.26	0.99	2.2819
	Chinese leymus grassland	0.67	60.03	2.50	0.85	2.9418
	Natural mowed grassland	0.66	60.07	2.50	0.85	2.9481

Ecosystems are open, with inputs and outputs of matter and energy. The pure natural ecosystem has no or negligible matter and energy input of human investment, but the artificial or semi-artificial ecosystem is more complex. There are not only structural differences among system types, but also obvious differences in functional status. As we can see from Table 1, the natural emergy inputs for 12 ecosystems were the same, to 5.31 × 10^14^ sej/ha/year [the unit of emergy is emJoule, a unit referring to the available energy of one kind consumed in transformations. Usually a unit of solar emergy expressed in solar emergy joules (abbreviated sej) is used to determine the value of environmental and human work within a system on a common basis]. But their average sum emergy inputs was more than 15 times different, and furthermore the average purchased emergy inputs was nearly 40 times the gap. It showed the rule roughly of “commercial crop > artificial forage > field crop > grassland”. Table 2 showed that from economic crops to artificial forage, to field crops, and grassland, emergy investment ratio (EIR) and environmental load (ELR) have a downward trend in turn, while the emergy self-sufficiency rate (ESR) and net emergy output rate (EYR) showed a tendency to increase obviously, and the emergy sustainability index (ESI) of the whole ecosystems increased significantly. This fully indicates that the higher the output of an ecosystem, the higher the human emergy that needs to be invested, and the greater the environmental load, the lower the sustainability. To some extent, natural resource emergy input is the basic power to maintain the operation of the ecosystem; purchased (artificial) emergy input is the fundamental cause of the ecosystem change under the same environmental conditions [33].

#### Emergy input changes of the same ecosystem type under different climatic conditions

A certain ecosystem is distributed in a certain environment, limited by the moisture and temperature conditions, there are different vegetation distribution belts from the equator to the poles of the earth. China has cold temperate, temperate, warm temperate, subtropical and tropical climates from north to south, and the vegetation is distributed in turn: the coniferous deciduous forest, temperate coniferous and broad-leaved mixed forest, warm temperate deciduous broad-leaved forest, north Asia subtropical deciduous broad-leaved forest with evergreen components, middle and south Asia tropical evergreen broad-leaved forest, tropical seasonal rain forest and rain forest. Nowadays, humans have dramatically transformed natural systems around the world as their capacity to manage their environment for multiple uses has evolved in step with to agricultural, industrial and green revolutions. Numerous natural ecosystems have been replaced by various semi-artificial or artificial ecosystems. The same ecosystem also appears even under different environmental conditions. For example, China’s two major crops, corn and wheat, have their footprints in almost every climate zone from south to north. Based on the statistical data of the main provinces of each climatic zone in 2014, we analyzed the emergy input of maize and wheat ecosystem, as showed in Tables 3 and 4.

**Table 3:** Natural and purchased emergy input in major wheat planting areas of China Some data refer to Zhao et al. [34]

Ecological zones	Natural emergy input (× 10^14^ sej/ha/year)	Purchased emergy input (× 10^14^ sej/ha/year)	Natural emergy input ratio (%)	Purchased emergy input ratio (%)
Huang-Huai-Hai Plain	5.91	36.95	14	86
Northwest of China	7.20	41.98	15	85
Loess Plateau	8.80	39.55	18	82
Southwest of China	9.13	25.24	27	73
Northeast of China	9.55	13.80	41	59

**Table 4:** Natural and purchased emergy input in major maize planting areas of China Some data refer to Zhai et al. [35]

Ecological zones	Natural emergy input (× 10^14^ sej/ha/year)	Purchased emergy input (× 10^14^ sej/ha/year)	Natural emergy input ratio (%)	Purchased emergy input ratio (%)
Northwest of China	8.32	47.19	15	85
Huang-Huai-Hai Plain	6.82	28.08	20	80
Loess Plateau	10.16	35.84	22	78
Southwest of China	11.48	35.65	25	75
Northeast of China	10.99	25.97	30	70

China has a broad geographical and diverse climate, the average yield is about 4500 kg/ha and 9000 kg/ha for wheat and maize respectively, but their input emergy are quite different based on the above table’s data. In the case of wheat ecosystem, the purchased emergy input in Northeast and Southwest of China accounts for about 59% and 73% of the total input respectively, while other areas are more than 80%; maize production is similar, these ratios are close to 70% to 75% in the Northeast and Southwest respectively, while in the Loess Plateau, the Huang-Huai Hai Plain and the Northwest area are 78–85%. Under normal conditions, the yield of maize and wheat mainly depends on the amount of natural and auxiliary energy input, and the input of auxiliary energy is closely related to the moisture and temperature conditions of each climatic zone and the soil fertility. Northeast China is rich in corn, wheat and soybeans, while North China and Northwest China are rich in wheat, and rice cultivation in the south is large. The so-called main crop producing area in China is a paradigm that humans have gradually explored in the long-term production practice to make full use of natural resources. In fact, it is an alternative to obtain high yield with less man-made (auxiliary) energy input.

### The mechanism of ecosystem evolution

Terrestrial ecosystem has relatively stable characteristics in a certain time and space range due to ecological resilience, which is the ability of a system to persist in the face of perturbations [22], but the life component in the ecosystem structure is changing all the time. When this change reaches a certain degree, or exceeds a certain “threshold” [36], the ecosystem functions also will have the fundamental change, finally causing the ecosystem evolution. This is a systematic evolution marked by community succession. The principle of ecosystem structure and function shows that structure is the basis of function, function is the embodiment of structure; change is absolute, and stability is relative. In the long history of the earth, the ecosystem experienced the changes from aquatic to terrestrial life, lower to higher organism, and grass to wood. In fact, it is a concrete manifestation of system structure and function changes. At present, many artificial ecosystems have been built according to human purpose and demand, such as farmland, artificial grassland, greenhouse, economic forest, aquaculture farm and many more. These ecosystems have a fundamental change in structure and function compared with the original ecosystem. In addition to partial use of natural resources, more of them are supported by artificial input energy. Like the artificial climate room, factory plant production (soilless cultivation), etc., have basically separated from the natural environment, completely relying on artificial input for maintenance.

**Fig. 4:**
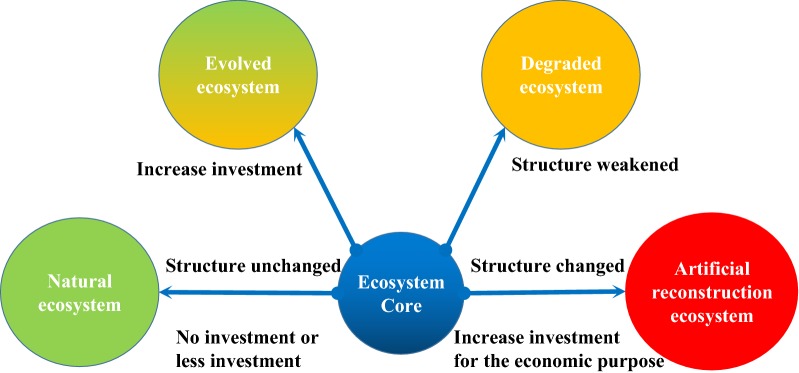
The mechanism of ecosystem evolution. “Ecosystem Core” refers to natural resources (energy)

Figure 4 shows that when the structure of the natural ecosystem becomes weaker, its function will also be degraded. For example, when the natural grassland is overused, the grassland degenerates and the function of production and biodiversity decrease [37]; when increasing input to the natural ecosystem, the function of the ecosystem can also be strengthened. For example, when fertilizing, irrigating, loosening soil, reseeding and other technical measures are carried out on natural grassland, the living environment of forage is improved, and the production capacity of grassland is obviously enhanced [38]. However, the two cases are without fundamental changes in the structure of their ecosystem, that is to say, within the scope of the “threshold” of the ecosystem, they belong to the ecosystem succession in the same location at different times.

The artificial reconstruction ecosystem is the structure of the natural ecosystem that has been partially or completely destroyed, and it also exhibits different system functions [39]. For example, natural grassland is reclaimed into farmland, the grazing function has become grain production. The essential driving force that determines this kind of ecosystem change is the economic purpose of mankind. It is the decision of input and output under the real economic and technological conditions, rather than absolutely following the principle of matter and energy input of the system. The fact of the large-scale use of chemical fertilizers and pesticides in the world today has also fully explained this point. Despite the fact that high production has been achieved, the natural environment has been severely damaged [40–42].

## Conclusion and perspective

“Ecosystem Core” hypothesis is an innovative explanation of ecosystem evolution, which is related to but distinct from the theory of community succession. Natural energy is the basic force for sustaining ecosystem development, and artificial energy input is the direct cause of ecosystem evolution. Different combinations of natural and purchased emergy are coupled to maintain the same ecosystem under the different environmental conditions. In general, certain natural resources correspond to specific natural ecosystems. With the disturbance of human energy, the natural ecosystem is separated from the original system development model and forms a new ecosystem. Ecosystem evolution should includes succession and reconstruction. The former is the functional evolution of the ecosystem without structural change, and the latter is a new ecosystem that is reconstructed according to human’s purpose and need. The evolution of an ecosystem is related to human economic purpose, its input–output ratio affects the goal of system reconstruction.

## Data Availability

The datasets in this study are available from the corresponding author on reasonable request.

## Abbreviations

Sej: solar emergy joules
EIR: emergy investment ratio
ELR: environmental loading ratio
ESR: emergy self-sufficiency ratio
EYR: emergy yield ratio
ESI: emergy sustainability index
